# Supplementary material for: Proteomic Analysis Identifies Dysregulated Proteins in Albuminuria: A South African Pilot Study
Source: Biology (Basel). 2024 Aug 30;13(9):680. doi: 10.3390/biology13090680 (PMC11428484; doi:10.3390/biology13090680)
Supplement: Supplementary file 1 [file biology-13-00680-s001.zip › biology-3148598-supplementary.pdf]

Supplementary for paper titled: Proteomic analysis identifies dysregulated proteins in albuminuria: a South African pilot study

Siyabonga Khoza [1,*], Jaya A. George [2], Previn Naicker [3], Stoyan H. Stoychev [4,5], June Fabian [6,7,†] and Ireshyn S. Govender [3,4,*,†]

[1] Department of Chemical Pathology, National Health Laboratory Service and School of Pathology, Faculty of Health Sciences, University of the Witwatersrand, Johannesburg 2000, South Africa
[2] Wits Diagnostic Innovation Hub, University of the Witwatersrand, Johannesburg 2000, South Africa
[3] Future Production Chemicals, Council for Scientific and Industrial Research, Pretoria 0001, South Africa
[4] ReSyn BioSciences, Edenvale 1610, South Africa
[5] Evosep Biosystems, 5230 Odense, Denmark
[6] Wits Donald Gordon Medical Centre, School of Clinical Medicine, Faculty of Health Sciences, University of the Witwatersrand, Johannesburg 2000, South Africa
[7] Medical Research Council/Wits University Rural Public Health and Health Transitions Research Unit (Agincourt), School of Public Health, Faculty of Health Sciences, University of the Witwatersrand, Johannesburg 2000, South Africa
* Correspondence: professor.khoza@wits.ac.za (S.K.); igovender@csir.co.za (I.S.G.)
† These authors contributed equally to this work.

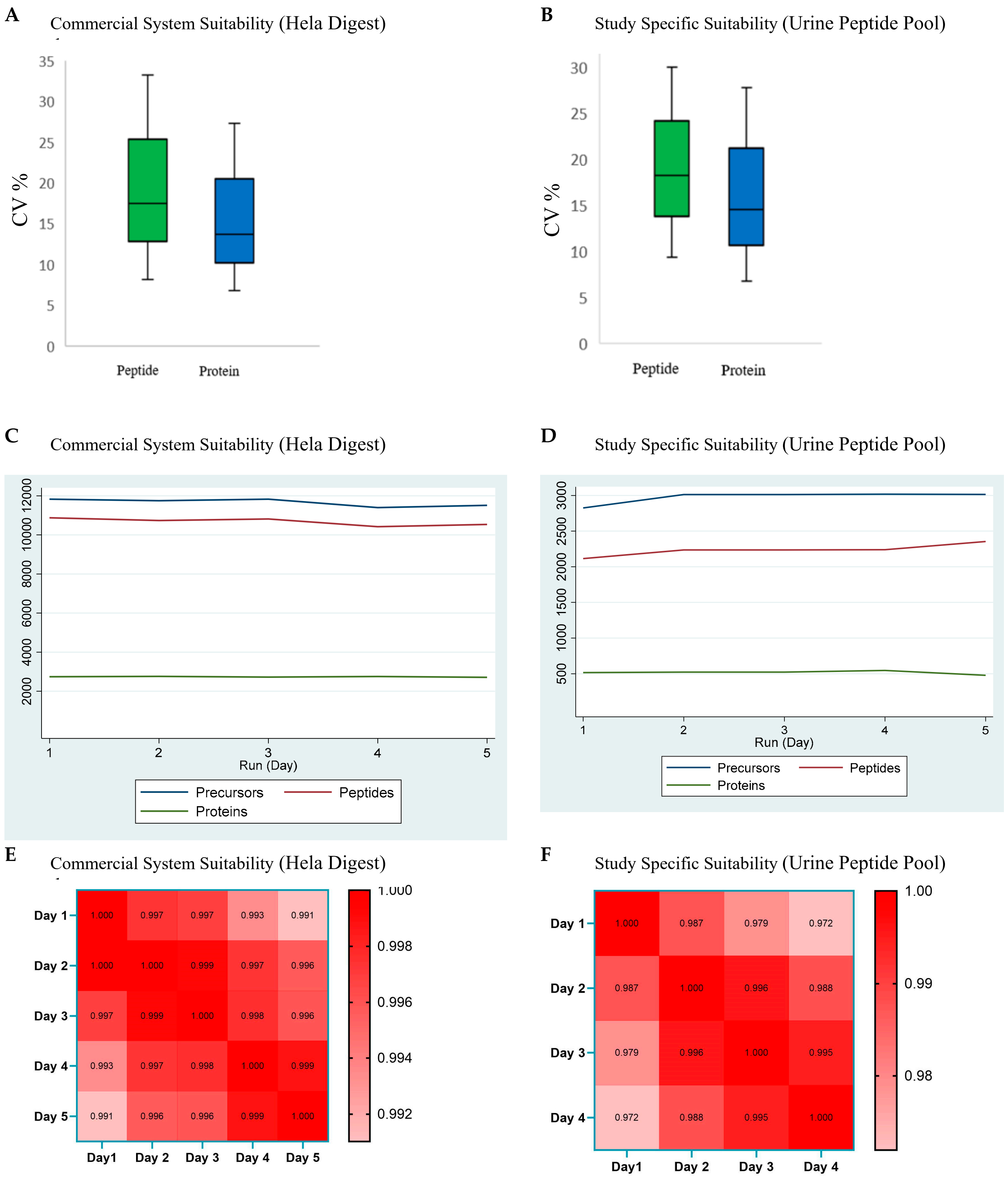

Figure S1 (A-F). Project specific system suitability and quality control (commercial Hela Digest and Urine peptide pool). CV, coefficient of variation.

Supplementary Table S1. Increased abundance dysregulated proteins in albuminuria sorted according to Q value

UniProt ID	Protein Descriptions	Protein Names	Q value	Mean Log2 fold change Ratio
P01009	Alpha-1-antitrypsin (SERPINA1)	A1AT_HUMAN	1.54E-34	2.904069
P04217	Alpha-1B-glycoprotein	A1BG_HUMAN	8.35E-32	2.655782
P02768	Albumin	ALBU_HUMAN	2.36E-31	3.103183
P43652	Afamin	AFAM_HUMAN	1.91E-30	2.56667
P01008	Antithrombin-III (SERPINC1)	ANT3_HUMAN	1.13E-24	2.749291
P02774	Vitamin D-binding protein	VTDB_HUMAN	1.10E-24	2.359199
P02144	Myoglobin	MYG_HUMAN	1.23E-20	3.631935
P02647	Apolipoprotein A-I	APOA1_HUMAN	2.53E-12	3.240776
P02679	Fibrinogen gamma chain	FIBG_HUMAN	2.96E-12	3.753340
Q96KN2	Beta-Ala-His dipeptidase	CNDP1_HUMAN	6.52E-11	2.696401
P02675	Fibrinogen beta chain	FGB_HUMAN	1.86E-11	2.494970
P04439	HLA class I histocompatibility antigen. A alpha chain	HLAA_HUMAN	1.90E-11	2.647121
P68871	Hemoglobin subunit beta	HBB_HUMAN	2.02E-11	3.501191
O43866	CD5 antigen-like	CD5L_HUMAN	2.14E-11	4.081572
P00915	Carbonic anhydrase 1	CAH1_HUMAN	2.76E-10	2.034928
P00747	Plasminogen	PLG	2.16E-10	1.499431
Q9UGM5	Fetuin-B	FETUB_HUMAN	1.98E-10	4.167315
P02747	Complement C1q subcomponent subunit C	C1QC	7.88E-10	2.812345
P01023	Alpha-2-macroglobulin	A2MG_HUMAN	4.89E-10	3.342841
P69891	Hemoglobin subunit gamma-1	HBG1_HUMAN	2.71E-08	3.246778
Q00610	Clathrin heavy chain 1	CLH1_HUMAN	5.40E-09	3.541298
A0A075B6K5	Immunoglobulin lambda variable 3-9	LV39_HUMAN	7.05E-09	2.414491
P80748	Immunoglobulin lambda variable 3-21	LV321_HUMAN	4.74E-08	2.960301
P61769	Beta-2-microglobulin	B2MG_HUMAN	1.54E-07	2.658679
P07148	Fatty acid-binding protein. liver	FABPL_HUMAN	1.82E-07	2.367101
P01714	Immunoglobulin lambda variable 3-19	IGLV3-19_HUMAN	7.07E-07	2.267423
P02654	Apolipoprotein C-I	APOC1_HUMAN	7.51E-07	1.815361
P04003	C4b-binding protein alpha chain	C4BPA_HUMAN	5.85E-07	3.398073
P02730	Band 3 anion transport protein	B3AT_HUMAN	5.49E-06	4.355263
Q969H8	Myeloid-derived growth factor	MYDGF_HUMAN	3.33E-06	3.335621
Q6UWP8	Suprabasin	SBSN_HUMAN	2.05E-06	3.065877
P30043	Flavin reductase (NADPH)	BLVRB_HUMAN	1.01E-05	2.497917
P11215	Integrin alpha-M	ITAM_HUMAN	4.14E-05	2.927536
P15090	Fatty acid-binding protein, adipocyte	FABP4_HUMAN	2.41E-05	4.667801
P04114	Apolipoprotein B-100	APOB_HUMAN	1.54E-05	2.706784
P24592	Insulin-like growth factor-binding protein 6	IBP6_HUMAN	5.06E-05	4.296296
P02753	Retinol-binding protein 4	RET4_HUMAN	3.97E-05	2.637620
P02042	Hemoglobin subunit delta	HBD_HUMAN	6.97E-05	3.390007

P15814	Immunoglobulin lambda-like polypeptide 1	IGLL1	2.95E-05	2.389023
P61026	Ras-related protein Rab-10	RAB10_HUMAN	2.16E-05	2.876893
P05023	Sodium/potassium-transporting ATPase subunit alpha-1	AT1A1_HUMAN	1.14E-05	2.453589
A0A087WW87	Immunoglobulin kappa variable 2-40	IGKV2-40_HUMAN	8.63E-04	3.105648
P60983	Glia maturation factor beta	GMFB_HUMAN	0.000043	3.737180
P07738	Bisphosphoglycerate mutase	PMGE_HUMAN	0.000227	4.363945
P50120	Retinol-binding protein 2	RET2_HUMAN	0.000385	2.965311
Q13231	Chitotriosidase-1	CHIT1_HUMAN	0.000388	4.775099
P62826	GTP-binding nuclear protein Ran	RAN_HUMAN	0.000572	2.644718
P02461	Collagen alpha-1(III) chain	CO3A1_HUMAN	0.000870	2.351171
P01611	Immunoglobulin kappa variable 1-12	KV112_HUMAN	0.001290	2.514485
P00568	Adenylate kinase isoenzyme 1	KAD1_HUMAN	0.002011	2.660483
P35579	Myosin-9	MYH9_HUMAN	0.002226	4.198412
P54578	Ubiquitin carboxyl-terminal hydrolase 14	UBP14_HUMAN	0.003342	3.308528
P06310	Immunoglobulin kappa variable 2-30	KV230_HUMAN	0.004164	2.861267
P06727	Apolipoprotein A-IV	APOA4_HUMAN	1.05E-07	3.525475
P24666	Low molecular weight phosphotyrosine protein phosphatase	PPAC_HUMAN	0.004316	2.687916
Q9NNX6	CD209 antigen	CD209_HUMAN	0.004914	2.723099
P02452	Collagen alpha-1(I) chain	COL1A1_HUMAN	0.00661	2.534567
P61626	Lysozyme C	LYZ_HUMAN	0.00548	2.745669
P00492	Hypoxanthine-guanine phosphoribosyltransferase	HPRT_HUMAN	0.005473	2.672041

Supplementary Table S2. Decreased abundance (high in normoalbuminuria group) dysregulated proteins in albuminuria group sorted according to Q value

UniProt ID	Protein Descriptions	Protein Name	Q value	Mean log fold change log 2 ratio
P01833	Polymeric immunoglobulin receptor	PIGR_HUMAN	9.96E-34	-2.555496
P12109	Collagen alpha-1(VI) chain	CO6A1_HUMAN	1.33E-30	-2.449243
P15941	Mucin-1	MUC1_HUMAN	7.87E-18	-2.265978
P39059	Collagen alpha-1(XV) chain	COFA1_HUMAN	1.99E-15	-2.689387
O75144	ICOS ligand	ICOSL_HUMAN	7.92E-15	-2.438606
P08572	Collagen alpha-2(IV) chain	CO4A2_HUMAN	6.32E-12	-2.802167
P07911	Uromodulin	UMOD_HUMAN	3.03E-11	-2.372999
P32942	Intercellular adhesion molecule 3	ICAM3_HUMAN	3.28E-10	-3.838267
P10451	Osteopontin	OSTP_HUMAN	1.89E-09	-2.295199
P16284	Platelet endothelial cell adhesion molecule	PECA1_HUMAN	2.42E-09	-2.874538
Q9NZD2	Glycolipid transfer protein	GLTP_HUMAN	2.57E-09	-3.375597
Q7Z5N4	Protein sidekick-1	SDK1_HUMAN	4.12E-09	-2.274248
P61970	Nuclear transport factor 2	NTF2_HUMAN	3.82E-07	-2.433140
P22307	Sterol carrier protein 2	SCP2_HUMAN	4.66E-07	-3.897821
P09488	Glutathione S-transferase Mu 1	GSTM1_HUMAN	0.000222	-2.823946
P30040	Endoplasmic reticulum resident protein 29	ERP29_HUMAN	0.000288	-4.255107
P32004	Neural cell adhesion molecule L1	L1CAM_HUMAN	0.000411	-3.717003
Q14116	Interleukin-18	IL18_HUMAN	0.002014	-2.295583
P08729	Keratin. type II cytoskeletal 7	K2C7_HUMAN	0.003037	-2.314029
P12814	Alpha-actinin-1	ACTN1_HUMAN	0.004721	-3.424070
Q96P63	Serpin B12	SPB12_HUMAN	0.005075	-2.591751

Supplementary Table S3. ROC curve analysis of all differentially abundant proteins

Protein name	Area under curve	P value
A1AT	0.95137931	2.90E-22
ALB	0.936896552	1.26E-19
ANT3	0.920689655	1.54E-17
AFM	0.903448276	1.22E-15
PIGR	0.879137931	1.65E-12
A1BG	0.85862069	1.76E-12
COL6A1	0.842758621	8.41E-11
MYG	0.831724138	3.86E-09
LV39	0.826724138	9.16E-10
MUC1	0.813103448	3.04E-09
ICOSLG	0.809137931	7.22E-09
UMOD	0.807413793	2.27E-08
VTDB	0.79862069	1.79E-08
COL15A1	0.778793103	1.78E-06
CAH1	0.776724138	1.28E-07
LV321	0.770689655	4.03E-07
PLMN	0.768448276	1.75E-06
CNDP1	0.756724138	3.48E-06
OSTP	0.746896552	3.86E-06
APOA4	0.741896552	2.48E-05
PECAM1	0.733448276	8.62E-06
NUTF2	0.731206897	3.71E-05
APOC1	0.727241379	3.11E-05
B2MG	0.724310345	0.000127475
FETUB	0.719310345	1.55E-05
KV112	0.714137931	3.90E-05
SDK1	0.705172414	0.000101757
CATZ	0.701896552	0.002150739
APOA1	0.688793103	9.53E-05
KRT7	0.688275862	0.045062916
B3AT	0.68	0.001123533
KV230	0.678275862	0.051773527
CD5L	0.677241379	0.001015201
RAB10	0.649310345	0.007830665
HLAA	0.647931034	0.002391733
HBB	0.644137931	0.00165894
PIMT	0.64362069	0.002323953
ERP29	0.642586207	0.013228896
FIBG	0.633965517	0.003786901
CLH1	0.627586207	0.005692606
KAD1	0.625689655	0.008093839
IBP6	0.625689655	0.00808161

FIBB	0.624310345	0.008266953
PMGE	0.621034483	0.008270821
HBA	0.619827586	0.009591786
IL18	0.619655172	0.181796271
GMFB	0.616551724	0.01036159
HPRT	0.612931034	0.024158448
KV240	0.612586207	0.085686393
COL4A1	0.609482759	0.015737301
CHIT1	0.607931034	0.06607194
HBD	0.607241379	0.03756034
C4BPA	0.603793103	0.0194972
HBG1	0.603793103	0.02539105
C1QC	0.601551724	0.028664713
CO3A1	0.595344828	0.195909359
L1CAM	0.595	0.088387058
FABPL	0.593965517	0.039587505
FABP4	0.593965517	0.033254869
A2MG	0.591724138	0.025853751
ACTN1	0.587758621	0.327328758
MYH9	0.587586207	0.131030419
APOB	0.584	0.031807543
RET2	0.575862069	0.018305553
UBP14	0.575344828	0.095410539
RET4	0.569827586	0.157470673
AT1A1	0.56637931	0.618572398
PPAC	0.562931034	0.336381777
SERPINB12	0.561206897	0.055967664
SCP2	0.560862069	0.402717537
SBSN	0.559827586	0.057042432
ITAM	0.555172414	0.698237266
CYTC	0.552758621	0.853321089
BLVRB	0.552413793	0.144938764
GSTM1	0.55137931	0.894527925
ICAM3	0.53862069	0.673757751
GLTP	0.530172414	0.743125431
RAN	0.522586207	0.727825673
CD209	0.512068966	0.617768845
MYDGF	0.504482759	0.353317965

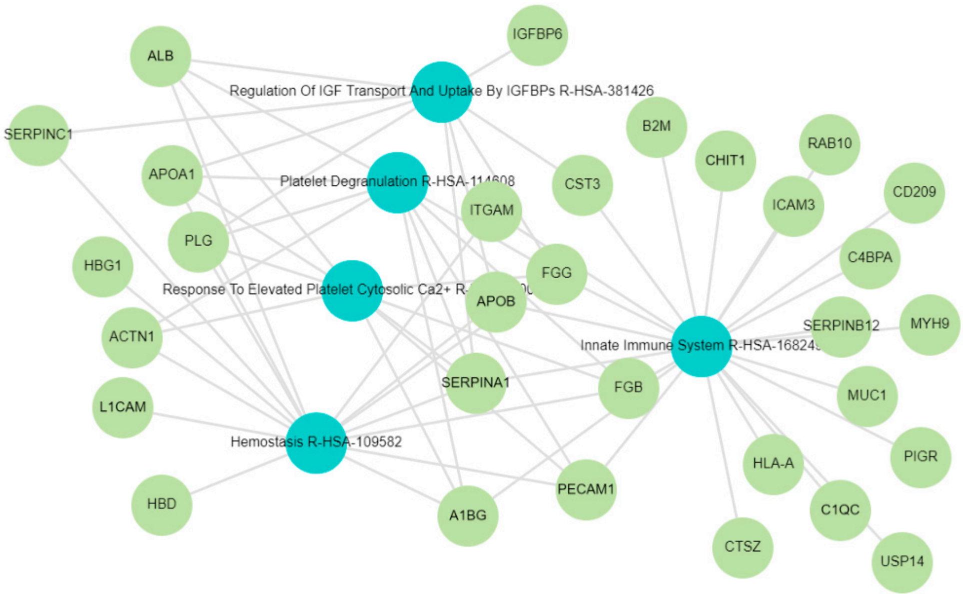

Figure S2. Network Analysis (Reactome library) through Enrichr-KG. Blue cycles indicates Reactome term. Green indicates genes involved.

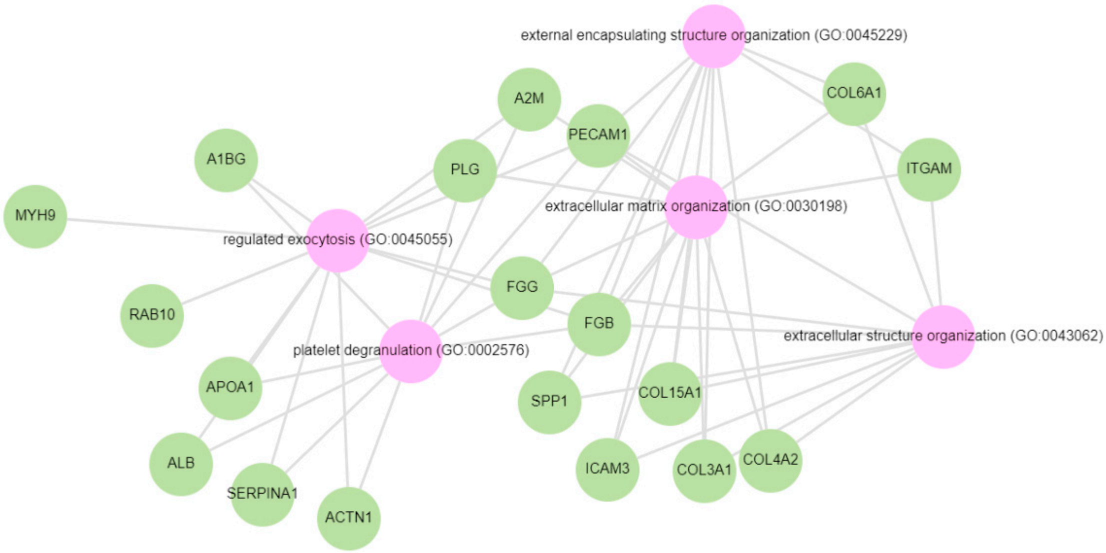

Figure S3. Enrichr-KG gene set enrichment analysis. GO biological-Enrich-KG: most of the proteins are involved in extracellular structure and matrix organization. Pink denotes GO biological process terms. Green denotes genes.

Supplematary Table S4: List of genes involved in the top 5 significantly enriched biological processes.

GO Biological Process	Genes	p-vale	q-value
Regulated exocytosis (GO:0045055),	PECAM1, FGB, A2M, SERPINA1, RAB10, ALB, MYH9, ACTN1, APOA1, A1BG, FGG, and PLG	6.91E-12	6.29E-09
Platelet degranulation (GO:0002576)	APOA1, A1BG, FGG, ALB, FGB, PECAM1, ACTN1, SERPINA1, PLG, and A2M	7.18E-11	3.27E-08
Extracellular matrix organization (GO:0030198)	COL6A1, A2M, PLG, COL4A2, FGG, PECAM1, SPP1, COL15A1, ITGAM, FGB, COL3A1, and ICAM3	2.53E-09	7.67E-07
Extracellular structure organization GO:0043062)	COL3A1, ITGAM, SPP1, ICAM3, COL6A1, COL15A1, COL4A2, PECAM1, FGG, and FGB	1.49E-08	2.65E-06
External encapsulating structure organization (GO:0045229)	COL6A1, COL15A1, ICAM3, ITGAM, SPP1, FGB, PECAM1, COL3A1, COL4A2, and FGG	1.56E-08	2.65E-06
